# Molecular and genomic typing for tuberculosis surveillance: A survey study in 26 European countries

**DOI:** 10.1371/journal.pone.0210080

**Published:** 2019-03-13

**Authors:** Marta Andrés, Marieke J. van der Werf, Csaba Ködmön, Stefan Albrecht, Walter Haas, Lena Fiebig, Survey study group

**Affiliations:** 1 Department of Infectious Disease Epidemiology, Robert Koch Institute, Berlin, Germany; 2 European Centre for Disease Prevention and Control (ECDC), Stockholm, Sweden; St Petersburg Pasteur Institute, RUSSIAN FEDERATION

## Abstract

**Background:**

Molecular typing and whole genome sequencing (WGS) information is used for (inter-) national outbreak investigations. To assist the implementation of these techniques for tuberculosis (TB) surveillance and outbreak investigations at European level there is a need for inter-country collaboration and standardization. This demands more information on molecular typing practices and capabilities of individual countries. We aimed to review the use of molecular/genomic typing for TB surveillance in European Union and European Economic Area countries in 2016; assess its public health value; and collect experiences on typing data use for cross-border cluster investigations.

**Method:**

A web-based questionnaire was provided to all TB National Focal Points. The questionnaire consisted of three parts: i) Use and integration of molecular and genomic typing data into TB surveillance; ii) Cross-border cluster investigation and international collaboration, and iii) Perception and evaluation of public health benefits of molecular and genomic typing for TB surveillance.

**Results:**

Of 26 responding countries, 20 used molecular typing for TB surveillance, including nine applying WGS. The level of integration into the national surveillance was heterogeneous. Among six countries not using typing for TB surveillance, more than half planned its implementation soon. Overall, most countries perceived an added public health value of molecular typing for TB control. Concerning international cluster investigations, countries had little experience and did not have standard protocols to exchange typing data.

**Conclusion:**

Our study shows a wide use of molecular and genomic typing data for TB surveillance in EU/EEA countries and reveals that transition to WGS-based typing is ongoing or is considered in most countries. However, our results also show a high heterogeneity in the use and integration of typing data for TB surveillance. Standardization of typing data use for TB surveillance is needed and formal procedures should be developed to facilitate international collaboration.

## Introduction

Molecular typing of *Mycobacterium tuberculosis* complex (MTB) is increasingly used to strengthen tuberculosis (TB) surveillance. 24-locus mycobacterial interspersed repetitive units variable number of tandem repeats (24 MIRU-VNTR) has become a standard tool [1, 2]. Yet, the transition to whole genome sequencing (WGS) is ongoing in the European Union (EU) and European and Economic Area (EEA) [3, 4]. Due to its higher discriminatory power [5] and potential to detect drug resistance [6], WGS is becoming a powerful tool to investigate TB outbreaks [7–10]. Recently, national TB contact points and reference laboratories supported by the European Centre for Disease Prevention and Control (ECDC) have used WGS-based typing to detect and clarify cross-border TB transmission [11, 12].

A recent review of the European Reference Laboratory Network for Tuberculosis (ERLTB-Net) [5], however, underlines that the appropriate role of WGS in TB surveillance remains to be defined and further evidence on the technical capacity across EU/EEA is needed before WGS-based surveillance for multidrug-resistant (MDR) TB can be operationalized [13, 14].

We have performed a questionnaire survey among EU/EEA Member States to i) review current practices in application of molecular/genomic typing for TB surveillance and capacity of transition to WGS-based typing; ii) explore the capability to use the typing in cross-border cluster investigations; and iii) assess its added public health value for TB surveillance, and to identify areas for future actions.

## Methods

A web-based questionnaire (Acuity 4 Survey, Voxco) was developed. It was piloted amongst five volunteering countries (Denmark, Italy, the Netherlands, Norway and Sweden) and thereafter adjusted.

The survey was conducted between September and November 2016 among TB National Focal Points of all EU/EEA Member States. Participants were encouraged to consult with other competent bodies in their country if needed. Countries not responding were followed up with two reminder emails.

The questionnaire consisted of three parts: i) Use and integration of molecular/genomic typing data into TB surveillance; ii) Cross-border cluster investigation and international collaboration, and iii) Perception and evaluation of public health benefits. The questionnaire comprised 23 closed- and three open-ended questions. Data protection was guaranteed by the Server architecture and the data protection concept of the RKI and approved by the data protection and legal departments of the RKI, resulting in a waiver for ethical review.

We performed a descriptive analysis of the collected data using Stata 14.0. Maps were generated using Regiograph (http://regiograph.gfk.com/).

## Results

**Fig 1:**
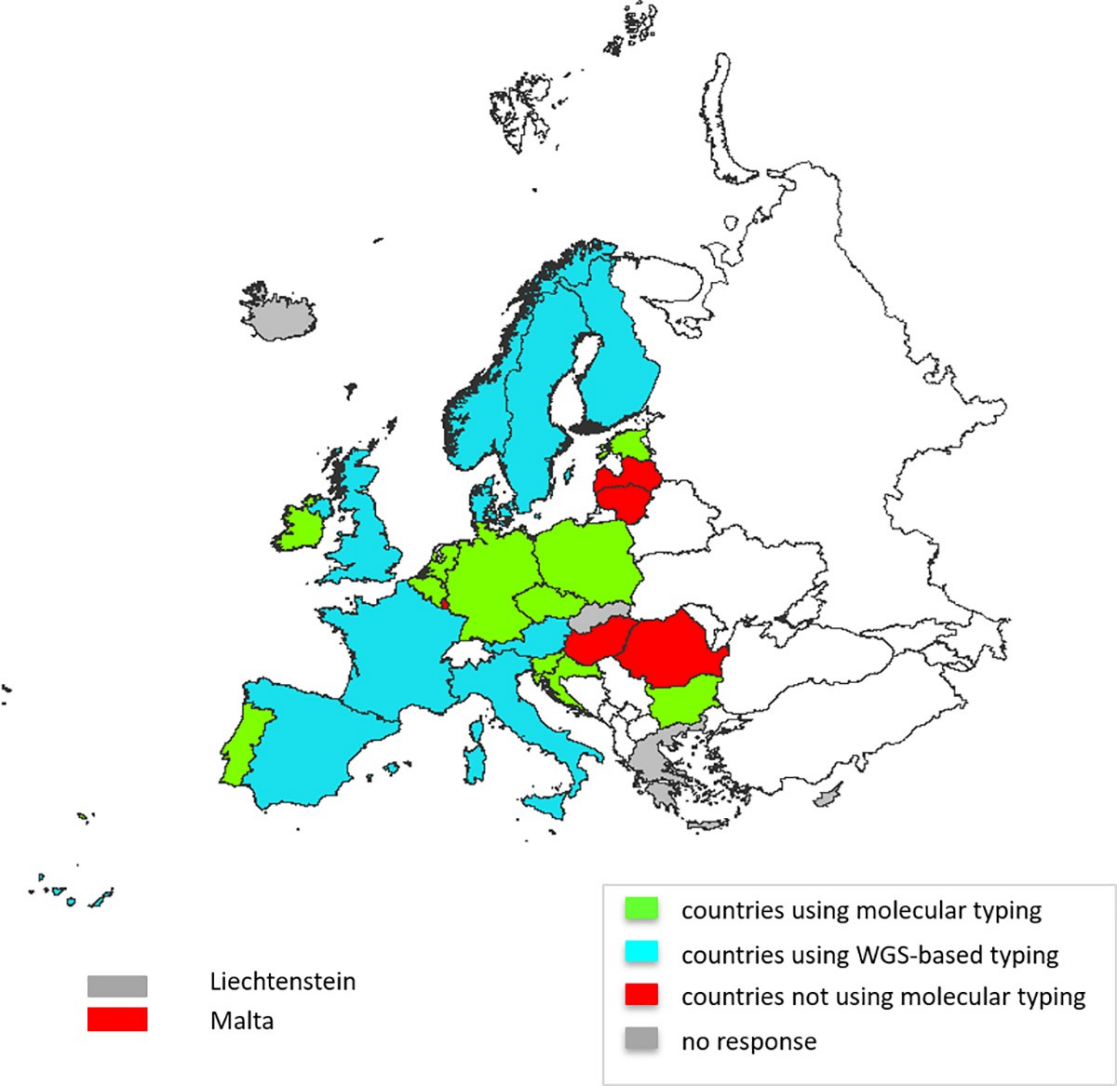
Molecular typing for TB surveillance in European Union/European Economic Area countries in 2016.

Of the twenty-six responding EU/EEA countries (26/31; 84% response rate), 20 countries did and six did not use molecular/genomic typing for TB surveillance (Fig 1).

### Countries using molecular/genomic typing for TB surveillance

Of the 20 countries that used typing data for surveillance, 19 used them for national surveillance and four also at sub-national level. Spain used typing data only at sub-national level (Table 1). 24 MIRU-VNTR was used by all countries. Seven countries used exclusively 24 MIRU-VNTR and 13 combined it with either spoligotyping (4/20); WGS (5/20); spoligotyping and WGS (3/20) or IS6110-RFLP, spoligotyping and WGS (1/20). Overall, nine countries (Austria, Denmark, Finland, France, Italy, Norway, Spain, Sweden and UK) used WGS, ten more countries considered its introduction (Table 1).

**Table 1:** Overview of integration of molecular typing methods in TB surveillance systems in European Union/European Economic Area countries.

Country	Molecular typing for surveillance (since year)	Administrative level	Methods used	WGS-based typing		Kind of isolates typed*	Coverage in 2015 (%)*		Typing laboratory*	Median reporting time (in days)*		Case-based integration*	Method outbreak investigations
				Use	(If no) Planned, year		MTB isolates	M/XDR isolates		spoligo	24 MIRU-VNTR		
Austria	Yes (2002)	National	Spoligo; 24 MIRU-VNTR; WGS	Yes	NA	MTB	90	100	NRL	10	30	Yes, national	Spoligo; 24 MIRU-VNTR; WGS
Belgium	Yes (2000)	Brussels region and specific groups nationally	Spoligo; 24 MIRU-VNTR	No	Yes, 2017^2^	MTB	40	100	NRL	30	30	Yes, national	Spoligo; 24 MIRU-VNTR
Bulgaria	Yes (2007)	National	Spoligo; 24 MIRU-VNTR	No	No	M/XDR-MTB	NA	68	NRL	250	250	Yes, national	None
Croatia	Yes (2007)	National	24 MIRU-VNTR	No	Yes	MTB	95	100	NRL	NA	30	Yes, national	24 MIRU-VNTR
Czech Republic	Yes (2010)	National	24 MIRU-VNTR	No	Yes, 2017–2018	MTB	95	100	NRL	NA	3	No	24 MIRU-VNTR
Denmark	Yes (1992)	National	24 MIRU-VNTR; WGS	Yes	NA	MTB	97	100	NRL	NA	14	Yes, national	24 MIRU-VNTR
Estonia	Yes (2003)	National	24 MIRU-VNTR	No	Yes	M/XDR-MTB; polyresistant strains	NA	100	NRL; Com. typ. serv. outside country	NA	365	Yes, national	24 MIRU-VNTR
Finland	Yes (2000)	National	Spoligo; 24 MIRU-VNTR; WGS	Yes	NA	MTB	100	100	NRL; Com. typ. serv. outside country	30	60	Yes, national	spoligo; 24 MIRU-VNTR; WGS
France	Yes (1995)	National, local	24 MIRU-VNTR; WGS	Yes	NA	M/XDR-MTB; outbreak	4	99	NRL; Com. typ. Serv.	NA	10	Yes, national	24 MIRU-VNTR
Germany	Yes	National	Spoligo; 24 MIRU-VNTR	No	Yes	M/XDR-MTB	Not known	90	NRL; PLL	Do not know	Do not know	No	spoligo; 24 MIRU-VNTR
Hungary	No Planned for 2017^1^		24 MIRU-VNTR	No	No								
Ireland	Yes (2011)	National, regional, local	24 MIRU-VNTR	No	Yes	MTB	93	100	NRL	NA	30	Yes, national, regional, local	24 MIRU-VNTR
Italy	Yes (2004)	National, regional, local	24 MIRU-VNTR; WGS	Yes	NA	M/XDR-MTB; outbreak (in some regions all MTB isolates)	Not known	53	NRL; RLL; Clin. Lab.; Research Institutes/Universities	NA	240	Yes; regional,local	24 MIRU-VNTR; WGS
Latvia	No		IS6110-RFLP; spoligo	No	Not known								
Lithuania	No (Planned 2017–2018)		24 MIRU-VNTR	No	Yes, 2017–2018								
Luxembourg	No (Planned for 2017)		None	No	Yes, 2017								
Malta	No		None	No	No								
Netherlands	Yes (1993)	National	24 MIRU-VNTR	No	Yes, 2018^2^	MTB	100	100	NRL	NA	14	Yes, national	24 MIRU-VNTR
Norway	Yes (1994)	National	24 MIRU-VNTR; WGS	Yes	NA	MTB	100	100	NRL	NA	14	Yes, national	24 MIRU-VNTR; WGS
Poland	Yes (2006)	National	spoligo; 24 MIRU-VNTR	No	Yes, 2018^2^	MTB	30	100	NRL	3	8	Not known	spoligo; 24 MIRU-VNTR
Portugal	Yes (2014)	National	24 MIRU-VNTR	No	Yes, 2016	M/XDR-MTB; outbreak	NA	96	NRL	NA	30	No	24 MIRU-VNTR
Romania	No (Planned for 2019)		None	No	Yes, 2019								
Slovenia	Yes (2000)	National	24 MIRU-VNTR	No	Yes, 2018^2^	MTB	100	100	NRL	NA	7	Yes, national	24 MIRU-VNTR
Spain	Yes	Regional, local	IS6110-RFLP; spoligo; 12 MIRU-VNTR; 24 MIRU-VNTR; WGS	Yes	NA	M/XDR-MTB; outbreak	Not known	Not known	NRL; PLL	Not known	Not known	Yes, regional, local	IS6110-RFLP; spoligo; 12 MIRU-VNTR; 24 MIRU-VNTR; WGS
Sweden	Yes (1996)	National	spoligo; 24 MIRU-VNTR; WGS	Yes	NA	MTB	95	100	NRL; clin. lab.	14	14	Yes, national	spoligo; 24 MIRU-VNTR; WGS
United Kingdom	Yes (2010)	National	24 MIRU-VNTR; WGS	Yes	NA	MTB	97	98	NRL; RRL	NA	65	Yes, national, regional, local	24 MIRU-VNTR; WGS

* Information in this table refers to spoligo- and 24 MIRU-VNTR. Information exclusively on WGS is provided in Table 2. Pink denotes countries not using molecular typing for TB surveillance, and therefore the questions are not applicable.

^1^ At the national level and for M/XDR-MTB isolates.

^2^ it is planned that 24 MIRU-VNTR will be simultaneously used.

Clin. Lab.: clinical laboratories; Com. typ. serv.: commercial typing service; MDR: multidrug-resistant tuberculosis; MIRU-VNTR: Mycobacterial Interspersed Repetitive Units - Variable Number of Tandem Repeat; MTB: Mycobacterium tuberculosis; NA: not applicable; NRL: National Reference Laboratory; PFGE: pulsed-field gel electrophoresis; PLL: peripheral level laboratories; rep-PCR: repetitive sequence-based-PCR; RFLP: Restriction fragment length polymorphism; RRL: regional reference laboratory; WGS: whole genome sequencing; XDR: extensively drug-resistant tuberculosis.

Of WGS-using countries, Austria used WGS as primary typing method (1/9) and five countries (Denmark, Finland, Italy, Norway and Spain) as secondary typing method to improve the resolution within spoligotyping and/or 24 MIRU-VNTR clusters (Table 2). Sweden was planning to use WGS as primary typing method in September 2016, and England (unknown for Wales, Northern Ireland & Scotland), Norway and Denmark from 2017. France was implementing progressively the use of WGS as high-resolution typing tool and Italy also used WGS for determination of drug-resistance.

**Table 2:** Overview of integration of WGS-based typing in TB surveillance systems in European Union/European Economic Area countries.

Country	Kind of isolates typed	Coverage		Typing laboratory	Use	Median reporting time (days)	Data analysis (cluster identification)	Case-based integration	Outbreak investigations
		MTB	M/XDR						
Austria	M/XDR; outbreak	10	50	NRL	Primary	15	Typing lab	No	Yes
Denmark	MTB	20	100	NRL	Secondary	14	Typing lab	No	No
Finland	M/XDR; outbreak	10	90	NRL	Secondary	30	Typing lab	No	Yes
France	M/XDR	NA	25	NRL; Com. typ. Serv.	Implemention in progress	30	Typing lab + surveillance unit	No	No
Italy	M/XDR; outbreak	2	53	NRL	Secondary	240	Typing lab	Yes; regional,local	Yes
Norway	Outbreak	Not known	Not known	NRL	Secondary	40	Typing lab	No	Yes
Spain	M/XDR; outbreak	Not known	Not known	NRL; PLL	Secondary	Not known	TB surveillance unit	Yes; regional,local	Yes
Sweden	MTB	30	100	NRL	Secondary (primary from September 2016 on)	14	Typing lab + surveillance unit	Yes	Yes
United Kingdom	In transition from 24 MIRU-VNTR for MTB	NA	NA	NRL	Implementation in progress.	NA	Set up in progress	No	Yes

Com. typ. serv.: commercial typing service; MDR: multidrug-resistant tuberculosis; MTB: *Mycobacterium tuberculosis*; NRL: National Reference Laboratory; PLL: peripheral level laboratories; RRL: regional reference laboratory; XDR: extensively drug-resistant tuberculosis; WGS: whole genome sequencing.

All 20 countries typed multidrug and extensively drug-resistant (M/XDR) TB isolates using 24 MIRU-VNTR. Average estimated typing coverage in 2015 was 95% (range 53–100). All but three countries (Bulgaria, Estonia and Germany) also systematically typed outbreak isolates and 13 countries typed all kinds of MTB isolates, eleven with a coverage of ≥ 90%.

WGS was used to type all MTB isolates in two countries (Denmark and Sweden); both M/XDR and outbreak isolates in four countries (Austria, Finland, Italy and Spain); only M/XDR isolates in France and only outbreak isolates in Norway. The UK was in transition from 24 MIRU-VNTR to WGS for all MTB isolates (Table 1). Three countries (Denmark, Finland and Sweden) typed ≥ 90% of their M/XDR isolates using WGS.

#### Where is typing performed?

All 20 countries had a National Reference Laboratory (NRL) that performed molecular/genomic typing. Additional typing laboratories included regional reference laboratories (2/20); peripheral level laboratories (2/20); clinical laboratories (2/20); research institutes (1/20) and a commercial typing service provider inside the country (1/20) or outside the country (1/20). All countries using WGS-based typing performed it in their NRL. In France and Spain, WGS-based typing was also performed by a commercial typing service provider and peripheral level laboratories, respectively.

The estimated median timespan between a MTB positive culture and reception of the typing results by surveillance units was 30 days for both 24 MIRU-VNTR (interquartile range, IQR, 14–60) and WGS (IQR 14–40).

#### Who is analyzing typing data?

Analysis of 24 MIRU-VNTR data to identify molecular clusters was performed by the typing laboratory (11/20); jointly by typing laboratory and surveillance units (7/20) or entirely by the surveillance unit (2/20).

Analysis of WGS data was mainly performed by the typing laboratory in Austria, Denmark, Finland, Italy and Norway, and jointly by typing laboratory and surveillance unit in France and Sweden, and by the TB surveillance unit in Spain. In England the analytical pipeline setup was in progress.

#### Integration of typing data into TB notification databases

Sixteen of 20 countries integrated the molecular/genomic typing data into the TB notification database on a case-based level. This integration occurred mostly at national level (14/16) and in Ireland and UK also sub-nationally. In Italy and Spain, integration only occurred sub-nationally. Only Italy and Spain integrated WGS results into a notification database (sub-nationally). Three countries (Czech Republic, Germany and Portugal) did not systematically integrate them, one country replied “unknown”.

### Countries not using molecular/genomic typing for TB surveillance

Six countries (Hungary, Latvia, Lithuania, Luxembourg, Malta and Romania) did not use molecular typing data for TB surveillance (Table 3). However, four of them considered its implementation soon (Hungary, Lithuania, Luxembourg and Romania) and three of them (Lithuania, Luxembourg and Romania) considered using WGS. Three countries (Hungary, Latvia and Lithuania) had performed molecular typing (mostly 24 MIRU-VNTR) of MTB isolates for research (3/6) or laboratory cross-contamination investigation (1/6).

**Table 3:** Overview of *M*. *tuberculosis* molecular typing practices in countries that do not use molecular typing for TB surveillance in European Union/European Economic Area countries.

Country	Molecular typing use	Molecular typing methods	Plan for molecular typing for TB surveillance	WGS plan as typing method?
Hungary	Research; Laboratory cross contamination investigation	24 MIRU-VNTR	Yes	No
Latvia	Research	IS6110-RFLP; spoligo	Not known	NA
Lithuania	Research	24 loci MIRU-VNTR	Yes	Yes
Luxembourg	No	None	Yes	Yes
Malta	No	None	No	NA
Romania	No	None	Yes	Yes

MIRU-VNTR: Mycobacterial Interspersed Repetitive Units - Variable Number of Tandem Repeat; NA: not applicable; NRL: National Reference Laboratory; PFGE: pulsed-field gel electrophoresis; PLL: peripheral level laboratories; rep-PCR: repetitive sequence-based-PCR; RFLP: Restriction fragment length polymorphism; RRL: regional reference laboratory; XDR: extensively drug-resistant tuberculosis; WGS: whole genome sequencing.

### Barriers for using molecular/genomic typing data in TB surveillance

Most countries (18/26) identified barriers for using typing data in TB surveillance (Table 4). “Financial constraints” was the most common barrier; both among countries using typing (10/20) and among countries not using it (5/6). Besides “financial constraints”, “human resources” (8/20) was most frequently mentioned by countries using molecular typing, while “data management and analysis” (3/6) by countries not using typing.

**Table 4:** Barriers for the use of molecular typing data for TB surveillance in European Union/European Economic Area countries.

	None	Do not know	Yes						
			Methodological (Lab) issues	Data management/analysis	Utilization of results for TB control	Human resources	Financial resources	Legal constraints	Not seen as a priority for TB surveillance
**Austria**	✓								
**Belgium**					✓	✓	✓		
**Bulgaria**				✓			✓		
**Croatia**	**✓**		** **	** **					
**Czech Republic**							✓		
**Denmark**						✓	✓		
**Estonia**^**1**^						✓	✓		
**Finland**					✓	✓	✓		
**France**	✓								
**Germany**				✓	✓	✓	✓	✓	
**Hungary**							✓		
**Ireland**	✓								
**Italy**^**2**^					✓		✓		
**Latvia**				✓	✓		✓	✓	
**Lithuania**							✓		✓
**Luxembourg**				✓		✓			
**Malta**							✓		
**Netherlands**					✓			✓	
**Norway**					✓	✓			
**Poland**	✓								
**Portugal**	✓								
**Romania**			✓	✓		✓	✓		
**Slovenia**	✓								
**Spain**			✓			✓	✓		✓
**Sweden**	✓								
**United Kingdom**					✓	✓	✓		
**Mol. typing countries**	40% (8/20)	0% (0/20)	5% (1/20)	10% (2/20)	35% (7/20)	40% (8/20)	50% (10/20)	10% (2/20)	5% (1/20)
**Non-mol. typing countries**	0	0	17% (1/6)	50% (3/6)	17% (1/6)	33% (2/6)	83% (5/6)	17% (1/6)	17% (1/6)

^1^ Other: Not enough experience to use molecular typing data in routine surveillance

^2^ Other: Lack of an agreed policy on utilising typing data for tracing contacts routinely and investigating possible sources of transmission; Delay in receiving MDR/XDR isolates from the peripheral laboratories to the Reference typing laboratory.

As to specific barriers to WGS-based typing (Table 5), mainly “financial constraints” were reported, (6/9 countries performing WGS and 11/17 countries not performing WGS). Countries using WGS also highlighted “human resources” (5/9) as significant barrier. Countries not using WGS underlined “data management and analysis” (10/17) as relevant barrier. Six countries (including countries using and not using WGS) did not perceive WGS-specific barriers.

**Table 5:** Barriers for the use of WGS-based typing data for TB surveillance in European Union/European Economic Area countries.

	None	Do not know	Yes						
			Methodological (Lab) issues	Data management/analysis	Utilization of results for TB control	Human resources	Financial resources	Legal constraints	Not seen as a priority for TB surveillance
**Austria**	✓								
**Belgium**			✓	✓	✓	✓	✓		
**Bulgaria**			✓	✓	✓	✓	✓		✓
**Croatia**			✓	✓	✓	✓	✓		✓
**Czech Republic**			✓			✓	✓		
**Denmark**	✓								
**Estonia**		✓							
**Finland**			✓	✓		✓	✓		
**France**						✓	✓		
**Germany**^**1**^				✓	✓	✓	✓	✓	
**Hungary**			✓	✓			✓		
**Ireland**				✓		✓	✓		
**Italy**					✓		✓		
**Latvia**			✓	✓		✓	✓	✓	
**Lithuania**		✓							
**Luxembourg**				✓		✓			
**Malta**							✓		
**Netherlands**				✓			✓	✓	
**Norway**					✓	✓	✓		
**Poland**	✓								
**Portugal**	✓								
**Romania**			✓	✓		✓	✓		
**Slovenia**	✓								
**Spain**			✓			✓	✓		✓
**Sweden**	✓								
**United Kingdom**				✓	✓	✓	✓		
**WGS using countries**	44% (4/9)	0	22% (2/9)	22% (2/9)	33% (3/9)	55% (5/9)	67% (6/9)	0	11% (1/9)
**Non-WGS using countries**	18% (3/17)	12% (2/17)	41% (7/17)	59% (10/17)	23% (4/17)	53% (9/17)	65% (11/17)	18% (3/17)	12% (2/17)

^1^ Other: currently lack of standardization of method, cluster definition, nomenclature, service structure for rapid assessment and communication of information

Most countries claimed that standardization of WGS data analysis and outbreak investigation should be improved and that collaboration and data sharing should be facilitated. Several countries mentioned that countries with WGS capacity could support other countries without capacity.

### Cross-border cluster investigation and international collaboration

Fourteen of the 26 responding countries had been contacted at least once by another EU/EEA Member State to participate in cross-border cluster investigations; only seven countries (Austria, Germany, Ireland, the Netherlands, Slovenia, Sweden and UK) had actively approached another EU/EEA country for international collaboration.

Six countries (Ireland, the Netherlands, Norway, Slovenia, Sweden and UK) had established standard operational procedures (SOPs) to perform national cluster investigations but none had a SOP for international investigations. Countries relied on the following legal basis for the international exchange of patient information in cross-border cluster investigation: Decision No. 1082/2013/EU of the European Parliament and of the Council (8/26) [15], the International Health Regulation and Implementation Act of the country (8/26), and their respective national law (4/26). Half of the countries (13/26) did not know which legal framework applied.

Eight countries reported barriers for cross-border cluster investigations, seven reported that there were none, eleven did not know. Main barriers were: “different levels of integration of molecular typing data” (6/8); “[lack of] standardization of molecular typing methodologies” (5/8); “reluctance to share personal data of patients” (4/8) and “legal constraints” (4/8; Table 6).

**Table 6:** Barriers for cross-border molecular cluster investigations in European Union/European Economic Area countries.

	No	Do not know	Yes							
			Different integration levels	Methodology standardization	Reluctance share personal data	Data management/analysis	Financial resources	Legal constraints	Lack of political commitment	Not seen as a priority for TB surveillance
**Austria**	✓									
**Belgium**			✓							
**Bulgaria**		✓					✓			
**Croatia**	✓									
**Czech Republic**		✓								
**Denmark**			✓	✓	✓		✓	✓	✓	✓
**Estonia**		✓								
**Finland**			✓	✓		✓				
**France**		✓								
**Germany**				✓		✓		✓		
**Hungary**		✓								
**Ireland**			✓		✓					
**Italy**								✓		
**Latvia**		✓								
**Lithuania**		✓								
**Luxembourg**		✓								
**Malta**		✓								
**Netherlands**			✓	✓	✓			✓		✓
**Norway**		✓								
**Poland**	✓									
**Portugal**		✓								
**Romania**	✓									
**Slovenia**	✓									
**Spain**	✓									
**Sweden**	✓									
**United Kingdom**			✓	✓	✓	✓	✓			
**mol. typing countries**	35%(7/20)	30% (6/20)	30% (6/20)	25% (5/20)	20% (4/20)	15% (3/20)	15% (3/20)	20% (4/20)	5% (1/20)	10% (2/20)
**Non-mol. typing countries**	17% (1/6)	83% (5/6)	0	0	0	0	0	0	0	0

### Public health benefits

**Fig 2:**
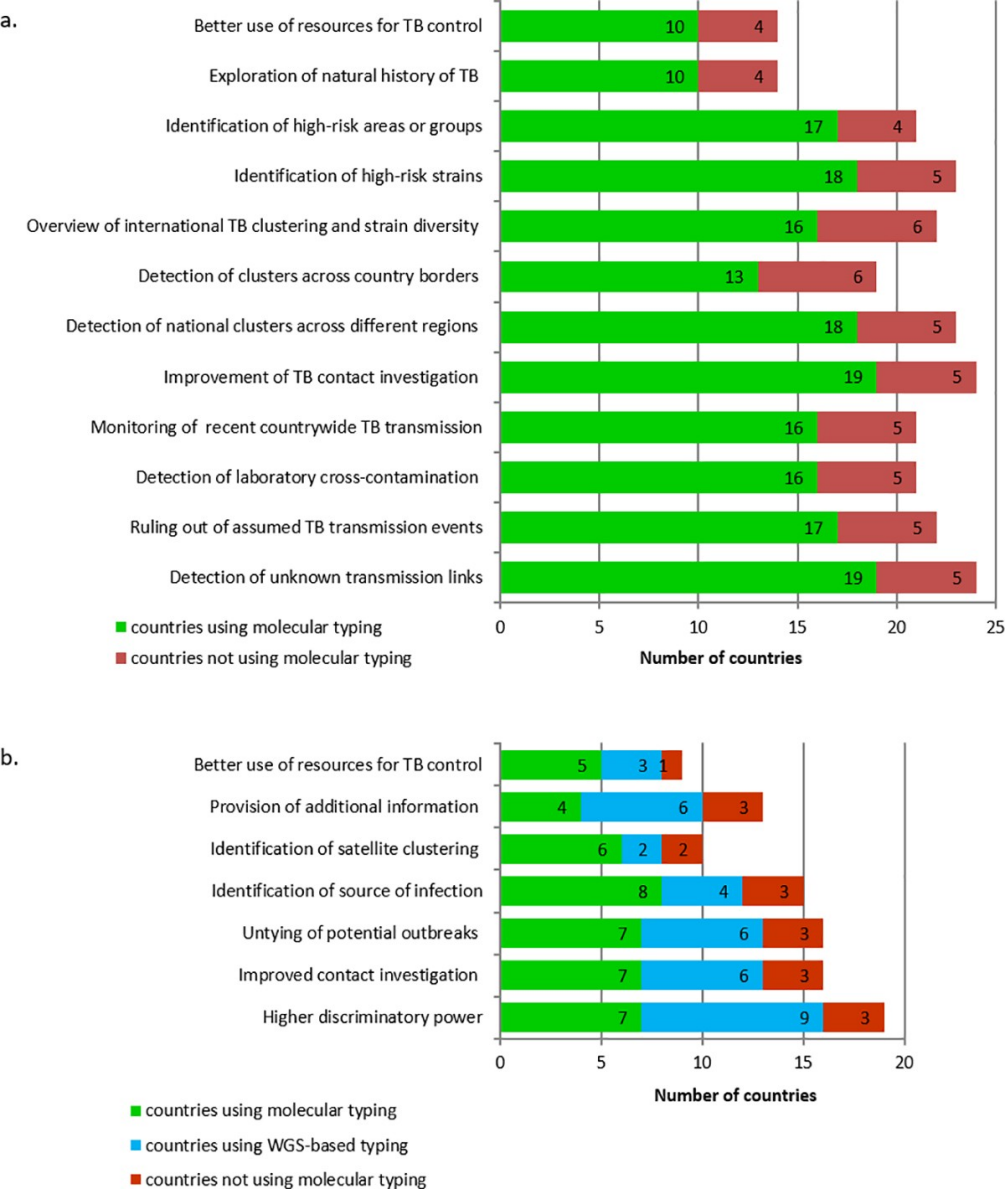
Added public health value of molecular typing (a.) and specifically WGS-based typing (b.) for tuberculosis in European Union/European Economic Area countries. Numbers in bars indicate number of countries.

All countries perceived a public health benefit of using molecular/genomic typing for TB surveillance for (Fig 2): 1. Detection of unknown transmission links (24/26, formally evaluated by 16 countries); 2. Improvement of contact investigation (24/26, formally evaluated by 13 countries); 3. Identification and investigation of high risk strains (23/26, formally evaluated by 14 countries) and 4. Detection of clusters across different regions (23/26, formally evaluated by 12 countries).

As to specific benefits of using WGS-based typing (Fig 2), 24/26 countries perceived a benefit, two countries did not know (Bulgaria and Malta). The main benefits were: 1. Higher discriminatory power (19/26); 2. Improved contact investigation (16/26) and 3. Untie potential outbreaks (16/26). Of the countries using molecular/genomic typing, most (19/20) considered that using WGS-based typing was beneficial, compared to two third (4/6) of the countries that did not use typing. WGS-using countries mostly considered WGS useful because it provides additional information, namely drug resistance.

## Discussion

Our survey shows that most EU/EEA Member States use molecular/genomic typing data for TB surveillance and the transition to WGS is ongoing. Our results also reveal substantial differences in the use and integration of typing data into national TB surveillance systems and identify financial constraints as the main barrier to a broader use, as well as limited experience in cross-border cluster investigation and a lack of respective SOPs. Most countries recognized a public health benefit of molecular typing and an additional benefit of using WGS-based typing.

The implementation of TB molecular surveillance is highly heterogeneous in EU/EEA countries in terms of the kind of typing laboratories, the selection of isolates, the coverage, the analytical approach used, and whether typing data are integrated into TB notification databases. In three countries typing results took ≥ 240 days to reach the surveillance units, which may limit the impact on TB control and is in contrast to the increasing speed of typing, e.g., in view of forthcoming genomic typing using direct samples [16].

Almost half of the responding countries that do not yet use molecular typing for TB surveillance, use it for other purposes such as diagnosis or research, and Hungary and Malta contribute to the ECDC MDR-TB molecular surveillance project while not using typing data for their national TB surveillance [17]. In Malta MDR-TB isolates are typed by the Dutch NRL [18] and in Hungary together by the Dutch and the Hungarian NRLs.

Overall, major barriers identified for using molecular typing data for TB surveillance were financial and human resource related. Countries using molecular typing also identified the “utilization of the data for TB control” as a barrier, e.g., clustering does not necessarily mean recent transmission [19–21] and therefore linking typing data to detailed epidemiological information of clustered cases is essential.

TB spread has an international dimension, as shown by the ECDC MDR-TB molecular surveillance project [17, 18]. WGS is being increasingly used to detect and clarify international outbreaks, such as an international MDR-TB cluster among asylum seekers that continues to expand across different EU countries [12], and a XDR-TB cross-border outbreak [22]. Rapid sharing of molecular/genomic typing data and epidemiological information between countries is important. Another investigation of a MDR-TB cluster in Austria, Romania and Germany using WGS [11] has demonstrated the need for establishing protocols for data sharing, which is supported by our results and previous studies [23]. However, EU/EEA countries still have limited experience on conducting cross-border cluster investigations and lack respective SOPs. This can potentially hamper international cluster investigations and subsequent measures of transmission control.

The major barriers for international collaboration were related to the different levels of typing data integration and insufficient standardization of molecular methodologies and data analysis. This may be even more complex when WGS-typing is used and exchanged given that data interpretation is more dependent on laboratory protocols and analysis pipelines [5, 24]. Therefore, standardized laboratory methodologies, analytical approaches and terminology are essential to ensure interchangeable among countries [25]. The finding that WGS-based typing is currently mostly exclusively performed by NRLs represents an opportunity for developing respective international standards before the laboratory network gets potentially more complex [14]. In 2017, ECDC initiated a pilot project on the use of WGS for molecular typing and characterization of *M*. *tuberculosis* in the EU and EEA. The project aims to standardize WGS laboratory procedures and bioinformatic analysis and to provide access to WGS for EU/EEA Member States that do not yet have capacity for WGS of *M*. *tuberculosis* [26].

None of the participating countries mentioned the quality and reliability of molecular/genomic typing results as a barrier to international collaboration. Proficiency testing of MIRU-VNTR typing has shown that laboratories face challenges with the inter- and intra-laboratory reproducibility of results [27]. The 2016 ECDC facilitated external quality assessment for 24 MIRU-VNTR typing showed that four of the 16 participating laboratories did not reach the threshold level for certification (unpublished data), which can critically compromise cluster investigations.

Demonstrating the added public health benefit of integrating molecular/genomic typing into TB surveillance systems remains challenging, [5] even though multiple scientific studies emphasize the power of molecular typing to clarify TB outbreaks [28, 29] particularly using WGS [7–10, 30]; monitor within country domestic transmission [31–33]; or identify high risk strains [34]. In our survey, surveillance units recognize the benefit of typing data for TB surveillance, especially for the detection of unknown transmission links and improvement of TB outbreak and contact investigations.

Strikingly, the countries not using molecular typing in-country mostly have a high percentage of MDR-TB cases [35]. And in several documented incidents, molecular typing performed abroad pointed M/XDR-TB transmission scenarios in these countries [11, 12]. This places even higher importance on timely international collaboration and information exchange, as well as on integrating these countries in molecular typing programs.

Previous economic evaluations of integrated molecular surveillance systems in England and the Netherlands suggested that the contribution of molecular typing to improve contact investigations is limited and the system was not cost-effective in the investigation period [36–38]. A recent study has shown the limited power of MIRU-VNTR to predict MTB genomic relatedness [39] but expects that the introduction of WGS-based typing may change this picture, given its higher discriminatory power and drug resistance detection [6, 40]. Further formal evaluations specifying and comparing different typing methods are thus needed, since different typing methods lead to different conclusions and demand different resources [40]. The area-wide introduction of a routine WGS-service by Public Health England offers a special opportunity to evaluate the added value and the costs of WGS-based typing for public health.

### Limitations

Our EU/EEA survey provides a general overview but may not replace in-depth technical exchange on integrated molecular TB surveillance systems. Despite the high response rate (84%), selection bias may not be entirely excluded, and our results may overestimate the current use of typing for TB surveillance in Europe. Since this is a rapid evolving field, some countries might have advanced in the implementation of WGS-based typing since the survey was performed, e.g., in the UK.

## Conclusions

Our study shows a wide use of molecular/genomic typing data for TB surveillance in EU/EEA countries and an ongoing transition to WGS-based typing. A high heterogeneity in their use and integration stress the need for timely standardization of WGS-based typing procedures and exchange of results, as well as administrative and legal frameworks and SOPs to facilitate international collaboration. The knowledge of pioneer countries and the perceived and observed public health benefits of molecular typing for TB control area favourable premise to tackle remaining challenges.
